# Increased Ndfip1 in the Substantia Nigra of Parkinsonian Brains Is Associated with Elevated Iron Levels

**DOI:** 10.1371/journal.pone.0087119

**Published:** 2014-01-24

**Authors:** Jason Howitt, Amanda M. Gysbers, Scott Ayton, Francine Carew-Jones, Ulrich Putz, David I. Finkelstein, Glenda M. Halliday, Seong-Seng Tan

**Affiliations:** 1 Florey Institute of Neuroscience and Mental Health, The University of Melbourne, Parkville, Australia; 2 Neuroscience Research Australia and the University of New South Wales, Sydney, Australia; St. Jude Children's Research Hospital, United States of America

## Abstract

Iron misregulation is a central component in the neuropathology of Parkinson's disease. The iron transport protein DMT1 is known to be increased in Parkinson's brains linking functional transport mechanisms with iron accumulation. The regulation of DMT1 is therefore critical to the management of iron uptake in the disease setting. We previously identified post-translational control of DMT1 levels through a ubiquitin-mediated pathway led by Ndfip1, an adaptor for Nedd4 family of E3 ligases. Here we show that loss of Ndfip1 from mouse dopaminergic neurons resulted in misregulation of DMT1 levels and increased susceptibility to iron induced death. We report that in human Parkinson's brains increased iron concentrations in the substantia nigra are associated with upregulated levels of Ndfip1 in dopaminergic neurons containing α-synuclein deposits. Additionally, Ndfip1 was also found to be misexpressed in astrocytes, a cell type normally devoid of this protein. We suggest that in Parkinson's disease, increased iron levels are associated with increased Ndfip1 expression for the regulation of DMT1, including abnormal Ndfip1 activation in non-neuronal cell types such as astrocytes.

## Introduction

Ubiquitination is a key process for the regulation of proteins in the cell and failure of ubiquitin pathways in the brain is linked to neuropathological states such as Parkinson's disease (PD) [1]. The targeting of proteins for ubiquitination relies on enzymes known as E3 ubiquitin ligases and their adaptor proteins; together they identify proteins for the addition of ubiquitin resulting in target protein degradation or alternatively, protein trafficking [2]. Ndfip1 is an adaptor protein for the Nedd4 family of ubiquitin ligases and has been found to be upregulated in neurons after brain injury, including head trauma, stroke and metal toxicity [3]–[6]. The upregulation of Ndfip1 is associated with binding and ubiquitination of a number of different protein substrates. One of these is the divalent metal transporter DMT1, which is targeted for ubiquitination and degradation in both the brain and liver in response to rising levels of transition metals [5], [7]–[9]. Specifically, Fe^2+^ and Co^2+^ can both stimulate increased Ndfip1 levels within primary human neurons in culture. This upregulation leads to a complex forming between Ndfip1, DMT1 and the ubiquitin ligase Nedd4-2, and results in the ubiquitination of DMT1 followed by its degradation [5]. The removal of DMT1 protects neurons from metal toxicity by limiting metal ion entry.

The present study was motivated by studies that report the involvement of DMT1 in the pathogenesis of PD [10]. PD is characterised by the degeneration of dopaminergic neurons in the substantia nigra and the accumulation of cytoplasmic Lewy body inclusions (containing α-synuclein, ubiquitin and iron) in these neurons [11]–[13]. However, PD is also directly associated with the intracellular accumulation of iron, particularly in the substantia nigra. These observations are strongly correlated with increasing severity of disease as reported in post-mortem histopathology and *in vivo* brain imaging [14]–[16]. Until recently the mechanism for this intracellular iron accumulation was unknown but new studies point to DMT1 misregulation as a primary cause [10], [17]. DMT1 can directly transport iron into the cell and is also required for iron exit from vesicles containing transferrin-bound iron [18]. Thus, DMT1 plays a critical role in regulating overall iron levels in the cell. In the brain, the abundance of DMT1 has been found to increase with age, suggesting a link between the transporter and metal misregulation in the development of age-based neurodegeneration. Consistent with this interpretation, postmortem PD brains contain more DMT1 compared to age-matched controls [10]. In animal studies, a direct link between DMT1 function and dopaminergic neuronal loss has been found. A spontaneous mutation in DMT1 found in both the *mk* mouse and Belgrade rat, results in deficits in iron transport. Experiments using both rodent mutants have shown that the animals are protected against experimentally induced PD using neurotoxins MPTP and 6-hydroxydopamine [10]. These results implicate a functional DMT1 gene with susceptibility to PD and a parsimonious interpretation would suggest that PD is linked to the failure of metal homeostasis.

The principle aim of this study was to identify changes in Ndfip1 expression in control and PD brains given that we have previously identified regulation of DMT1 by Ndfip1 [5]. To pursue this, we first studied the involvement of Ndfip1 in regulating DMT1 levels as well as cell survival during iron toxicity using mouse dopaminergic neurons. Secondly, we examined the levels of Ndfip1 and iron in the substantia nigra of PD brains and compared these with controls using biochemical analysis to identify changes in protein expression and metal concentrations. Thirdly, we compared the expression of Ndfip1 in different cells types using immunohistochemistry to identify the cells that upregulate Ndfip1 within the substantia nigra. Finally, we studied the expression of Ndfip1 with known markers of PD pathology to correlate the levels of Ndfip1 with neuronal stress. Our overall results show that Ndfip1 is upregulated in dopaminergic neurons and abnormally upregulated in astrocytes within the substantia nigra of PD brains, suggesting that Ndfip1 is responsive to the disease process and even abnormally activated in non-neuronal cells. Given the known role of Ndfip1 in regulating DMT1 and protecting neurons against stress, our results suggest that upregulation of Ndfip1 might represent attempts to regulate metal levels in PD pathogenesis.

## Methods

### Ethics statement

This study conformed to the Australian National Health and Medical Research Council's published Code of Practice for the Use of Animals in Research, and experiments were approved by the Florey Neuroscience Institutes animal ethics committee (#07-040). Human tissue experiments were approved by The University of New South Wales Human Research Ethics Advisory panel (#10075).

### Mesencephalic neuronal cultures and iron toxicity studies

The ventral mesencephalon was removed from embryonic day 13 (E13) of Ndfip1^+/+^ and Ndfip1^−/−^ embryos. Tissue was digested with the Papain Dissociation Kit (Worthington Biochemical Corporation, Lakewood, NJ, USA) and mechanically dissociated using a flamed tipped Pasteur pipette. Cells were plated at a density of 100,000 cells per 35 mm microwell plate on glass coverslips precoated with 0.5 mg/ml poly-D-lysine. Cells were maintained in serum-free Neurobasal medium supplemented with 0.5 mM glutamine, 2% B27 supplement (Invitrogen) and antibiotics (50 IU/mL penicillin and 50 µg/mL streptomycin). Experiments were performed after 7 days in vitro (DIV).

For iron toxicity studies cells were treated with FeCl_2_ containing 10 mM ascorbate oxidase to maintain the iron in a Fe^2+^ state. Cells were treated for 24 hours before fixing in 4% paraformaldehyde. For Ndfip1 immunocytochemistry coverslips were incubated overnight at room temperature (RT) in primary antibody (rat anti-Ndfip1 monoclonal antibody 1G5 [4]), 1∶500 in blocking buffer containing 10% fetal bovine serum and 0.1% v/v Triton X-100. Primary antibody signal was detected using an Alexa Fluor 594-congugated goat anti-rat antibody incubated for 1 hour at RT (1∶500 Invitrogen). To identify cell death TUNEL labelling was performed on each slide according to the manufacturer's instructions (Roche). Random fields from each coverslip were selected for imaging before analysis using ImageJ software. Total DAPI counts were performed (representing all neurons) along with total TUNEL counts (representing cell death) using the Analyse Particles function in ImageJ. The ratio of TUNEL/DAPI cells determined the percentage of dead cells for each field. Four fields per condition were analysed (∼100 cells for each sample) from 3 independent experiments.

### Tissue samples from human substantia nigra and cortex

Frozen and formalin-fixed brain tissue blocks from the substantia nigra and cingulate cortex were received from the Sydney Brain Bank and the NSW Tissue Resource Centre. These brain banks are part of the Australian Brain Bank Network funded by the National Health and Medical Research Council of Australia. Standardized clinicopathological criteria were used for diagnosis [19]. Tissue from 10 levodopa-responsive PD cases (5 aged 81±7 y with short disease durations of 4±4 y and 5 aged 81±9 y with end-stage disease of 16±1 y duration) that fulfilled the UK Parkinson's Disease Society Brain Bank Diagnostic Criteria [20] and had no other significant neuropathology were provided. Control tissue was matched (87±9 y, not different in post-mortem delay of 24±19 h or sex-distribution of 2–3 males and 2–3 females per group) from longitudinally-followed, community-dwelling brain donors who had no significant neuropathology and no evidence of neurological or psychiatric disease.

### Brain lysates and Western blotting

Frozen tissue samples were lysed in RIPA buffer (50 mM Tris, pH 7.2, 0.15 M NaCl, 2 mM EDTA, 1% NP-40, and 0.1% SDS) with protease inhibitor cocktail (Complete, Mini; Roche) for 25 min at 0°C. Brain homogenates were cleared of insoluble debris by centrifugation at 13,000 rpm for 15 min at 4°C. Protein concentration of lysates was measured using the detergent-compatible protein assay according to the manufacturer's instructions (Bio-Rad Laboratories). Lysates were resolved on 10% SDS-PAGE gels followed by transfer onto Hybond-C nitrocellulose membrane (GE Healthcare). Membranes were blocked for 1 h at RT in 5% nonfat milk in TBS and 0.05% Tween 20. Blots were incubated overnight with primary antibodies at 4°C followed by appropriate HRP-conjugated secondaries for 1 h at RT. Proteins were detected using ECL reagent according to the manufacturer's instructions (GE Healthcare) and visualized by exposure to x-ray film. The primary antibodies used were rabbit polyclonal Ndfip1 [5] and mouse monoclonal β-actin (Sigma). The HRP-conjugated secondary antibodies used were goat polyclonal anti–rabbit (1∶10,000; Millipore), and goat polyclonal anti–mouse (1∶15,000; Millipore). The level of Ndfip1 was calculated relative to the level of β-actin and is expressed as the Ndfip1/β-actin ratio.

### Metal analysis

Frozen tissue samples were washed three times in PB buffer. Tissue was digested in 70% nitric acid at 110°C and the iron level was determined by ICP-MS as described previously [21]. Cellular metal levels were normalized for total tissue weight and are expressed as the percentage difference from control levels.

### Immunohistochemistry

Formalin-fixed tissue blocks were cryoprotected in 30% buffered sucrose for 2 days and 20 µm thick serial sections cut on a cryostat and collected onto gelatinized slides for routine peroxidase immunohistochemistry and double-labelling immunofluorescence. Sections were first defatted in graded ethanols to xylene and rehydrated back through the graded ethanols to water. To help the antibody penetrate into the tissue and eliminate endogenous peroxidase activity, three 5-min 50% ethanol washes were performed, followed by incubation with 5% hydrogen peroxide in 50% ethanol for 40-mins.

For immunoperoxidase, sections were incubated overnight at RT with anti-Ndfip1 (rabbit polyclonal [5]) or anti-DMT1 (Alpha diagnostics NRAMP22-A) prepared in 1% normal horse serum block. After three 5-min washes in 0.1 M Tris buffer, sections were incubated in biotinylated secondary antibody for 1 hour at RT and followed by avidin–biotinylated horseradish peroxidase complex (ABC Elite kit; Vector PK-6100) for 30 min. The peroxidase labelling was visualized with Nickel enhanced DAB and the sections were washed in 0.1 M Tris buffer, dehydrated through graded ethanols to xylene and coverslipped using DPX (Sigma 317616). Images were captured using a Zeiss Axioskop 2 microscope. The number of pigmented neurons with and without Nidfip1 immunoreactivity in the entire substantia nigra was counted for each case and the proportion of pigmented neurons containing Ndfip1 immunoreactivity calculated. There was no significant difference in the numbers of neurons counted between two raters analysing 50% of the tissue sections.

For fluorescent double-labelling, sections were again defatted and rehydrated. Antigen retrieval was used to unmask α-synuclein (90% formic acid for 2-min) and GFAP (boiling in 10 mM sodium citrate buffer, pH 6.0, at 800 W in the microwave for 10-min). Sections were incubated with 5% hydrogen peroxide in 50% ethanol for 1 hour to eliminate endogenous peroxidase activity. After blocking with 1% BSA containing 0.02% Tween-20 for 1 hour, sections were incubated overnight at RT with either rabbit polyclonal anti-Ndfip1 and mouse monoclonal anti-α-synuclein (clone MAB42, BD Transduction Laboratories 610787) or rabbit polyclonal anti-Ndfip1 and mouse monoclonal anti-GFAP (Sigma G3893) prepared in 1% BSA block without Tween-20. After three 5-min washes in PBS, sections were incubated with either anti-rabbit HRP (Invitrogen T20924, component C; for Ndfip1) or Alexa fluor 488 anti-mouse (Invitrogen A11001; for α-synuclein and GFAP) secondary antibodies for 1 hour at RT and followed by three 5-min washes with PBS. For tissue subjected to anti-rabbit HRP, sections were incubated for a further 10-min at RT with Alexa fluor 568 tyramide (Invitrogen T20924, component A). All sections were washed in PBS and coverslipped, using Vectashield mounting medium for fluorescence (Vector Laboratories H-1000) and the coverslips sealed with nailpolish. Fluorescent images were captured using a Nikon Confocal Microscope ECLIPSE 90i. The proportion of neurons containing α-synuclein-immunoreactive inclusions that also contained Ndfip1 immunoreactivity was assessed. There was no significant difference in the proportion of double-labelled neurons between two raters analysing 50% of the tissue sections.

### Statistical analyses

Statistical analyses were performed using SPSS (IBM SPSS Statistics 18) and Graphpad Prism with *p* value less than 0.05 accepted as significant. Averages and standard deviation are given for all values of human PD brains. Group comparisons were performed using Mann Whitney U tests and correlations between variables were performed using Spearman Rho tests.

## Results

### Genetic loss of Ndfip1 in mouse dopaminergic neurons resulted in increased DMT1 and susceptibility to iron toxicity

**Figure 1:**
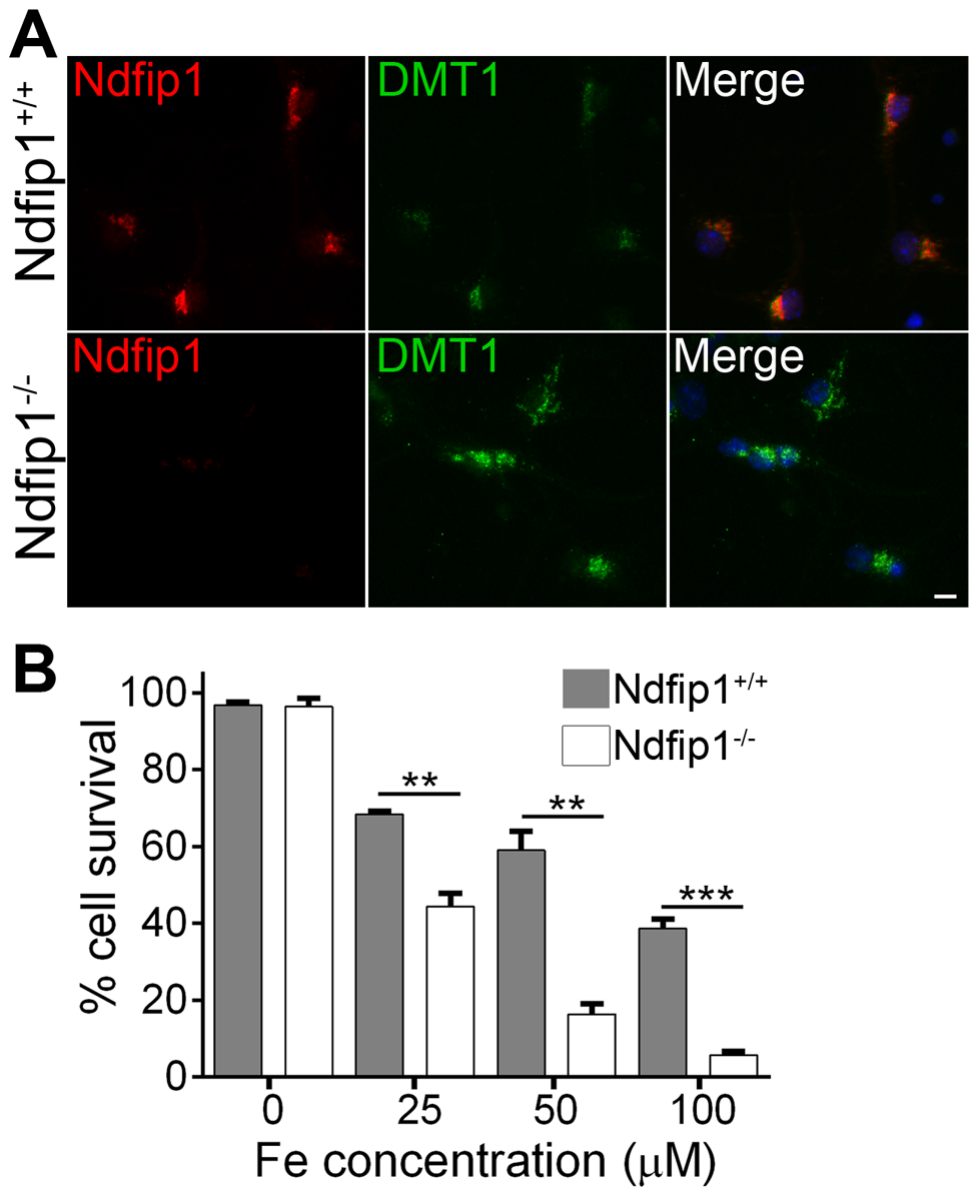
Loss of Ndfip1 results in misregulation of DMT1 and increased susceptibility to iron induced death. (A) Staining of mouse dopaminergic neurons in culture showed that genetic deletion of Ndfip1 (Ndfip1^−/−^) resulted in increased DMT1 staining compared to wild type (Ndfip1^+/+^) dopaminergic neurons. (B) Increasing concentrations of iron resulted in a significant increase in dopaminergic neuronal cell death in Ndfip1 knockout neurons compared to wild type controls. Values are means ±SEM, ** p<0.005, *** p<0.001 un-paired Student t-test. Scale bar: 10 µm.

To investigate the relationship between Ndfip1 levels and DMT1 regulation we studied dopaminergic neurons from the mid-brain of control and Ndfip1 knockout mice. Using immunocytochemistry we observed that loss of Ndfip1 from dopaminergic neurons resulted in increased levels of DMT1 compared to WT controls (Figure 1A). To determine if increased levels of DMT1 in Ndfip1 knockout neurons had an effect on cell survival during iron induced toxicity, we performed cell death assays using TUNEL labelling. Increasing concentrations of iron were observed to increase cell death in WT dopaminergic neurons consistent with previous reports (Figure 1B). Loss of Ndfip1 was observed to significantly increase the death of dopaminergic neurons undergoing iron induced stress compared to the WT controls (Figure 1B). Together our results suggested that Ndfip1 levels are important for the regulation of DMT1 in dopaminergic neurons and loss of this function resulted in cells that are hypersensitive to iron toxicity.

### Ndfip1 levels and iron concentrations are increased in the substantia nigra of PD brains

**Figure 2:**
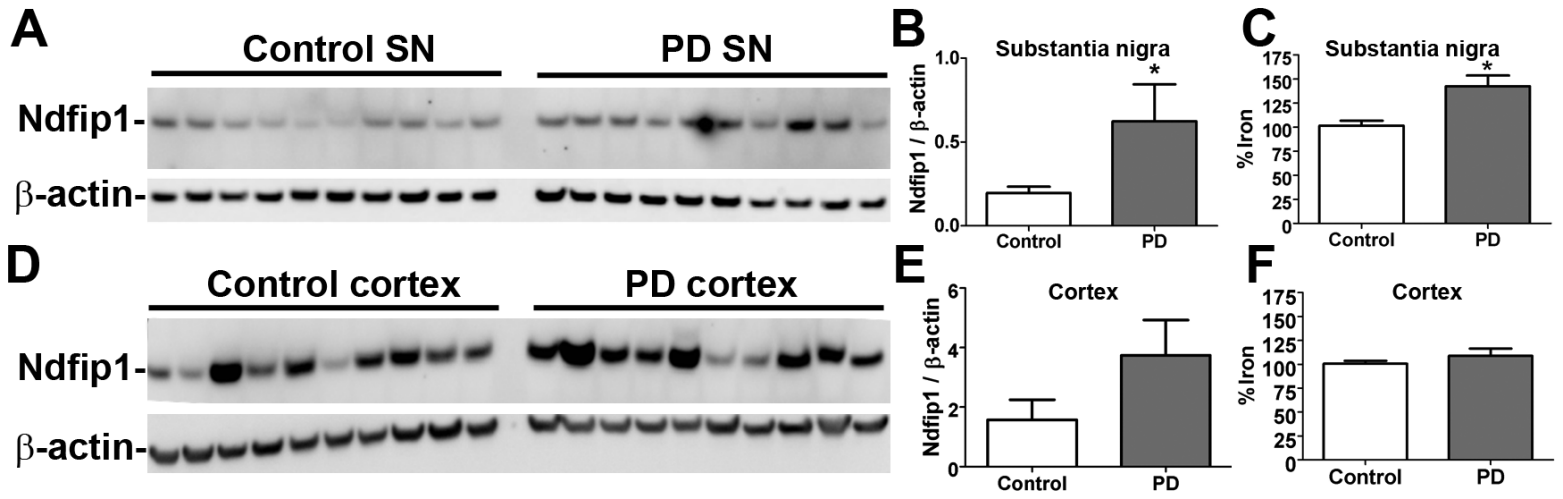
Ndfip1 is upregulated in the substantia nigra of PD brains. (A) Western blotting for Ndfip1 in the substantia nigra (SN) in both control and PD brains. (B) Quantification of Ndfip1 levels showed a significant increase of Ndfip1 in the substantia nigra of PD brains. (C) Iron content was analysed using ICP-MS on brain tissue from the substantia nigra of control and PD brains, a significant increase in iron levels in PD brains was observed. (D) Western blotting of Ndfip1 levels in the cortex of both control and PD brains. (E) Quantification of Ndfip1 levels showed no significant increase in Ndfip1 in the cortex in PD. (F) ICP-MS analysis for iron levels in the cortex showed no increase in iron concentrations between control and PD brains. (B, C, E, F) Values are means ±SEM, * p<0.05 un-paired Student t-test.

To investigate the regulation of Ndfip1 in Parkinson's disease brains the substantia nigra and cortex of 10 PD and 10 aged matched controls samples were analysed for Ndfip1 using Western blotting (Figure 2). All samples were found to have Ndfip1 expression to varying degrees, indicating that the protein is endogenously expressed in both the cortex and also in the substantia nigra of human brains. In PD brains, a significant increase in the levels of Ndfip1 was found in the substantia nigra compared to controls, after normalization to the β-actin control (Figure 2A, B). Total iron concentrations were also analysed using ICP-MS and, consistent with previous reports [22], [23], a significant increase in the level of iron was found in the substantia nigra of PD brains compared to control brains (Figure 2C). In the cortex, no significant increase in Ndfip1 levels were found in PD brains compared to control brains (Figure 2D, E), likewise the concentration of iron was unaltered between PD and control cortex lysates (Figure 2F). Together, these results show an upregulation of Ndfip1 in the substantia nigra in PD brains that is correlated with increased iron concentrations.

### Ndfip1 is found in dopaminergic neurons of the substantia nigra and increased Ndfip1 expression is correlated with α-synuclein deposits

**Figure 3:**
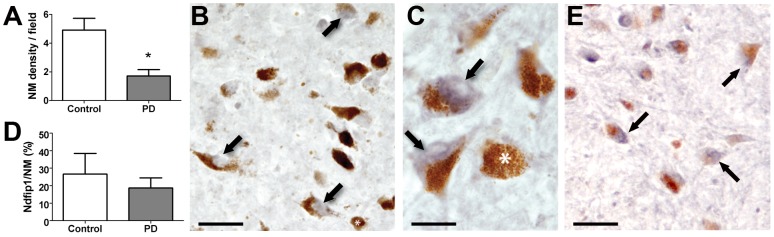
Ndfip1 and DMT1 are expressed in dopaminergic neurons of the substantia nigra. (A) Quantification of neuromelanin levels from the substantia nigra of control and PD brains showed a significant decrease in PD, indicating a loss of dopaminergic neurons. (B) Ndfip1 is expressed (arrows) in some but not all dopaminergic neurons of the substantia nigra (brown pigment represents neuromelanin). (C) High power image of Ndfip1 staining of dopaminergic neurons showed a predominant cytoplasmic distribution. Asterisks in B and C show dopaminergic neurons with no Ndfip1 staining. (D) Quantification of Ndfip1 positive dopaminergic neurons of the substantia nigra showed no significant difference between control and PD brains. (E) DMT1 is expressed in the cytoplasm (arrows) of dopaminergic neurons of the substantia nigra. Scale bars: 100 µm (B, E), 25 µm (C). (A, D) Values are the mean ±SD, * p<0.05 Mann Whitney U tests.

Our biochemical results suggested that Ndfip1 levels were increased in the substantia nigra of PD brains, however it was not clear which cell types in the substantia nigra have upregulated Ndfip1 levels. In rodents, Ndfip1 is exclusively expressed in neurons throughout the brain and under normal conditions is not found in astrocytes or oligodendrocytes. We therefore investigated the expression of Ndfip1 in dopaminergic neurons of the substantia nigra using fixed tissue from both control and PD brains. Counts of neurons containing neuromelanin confirmed that the PD brains had lost significant numbers of dopaminergic neurons (average 65±9% loss from control values, t = 2.73, p = 0.018) (Figure 3A) and as expected, there was significantly more loss with increased disease progression (t = 20, p<0.001). Immunohistochemical staining for Ndfip1 indicated that the protein was expressed in dopaminergic neurons with a predominant distribution in the cytoplasm (Figure 3B, C). However, not all dopaminergic neurons, in PD or control brains, were found to express Ndfip1. Counts of Ndfip1-positive-neurons indicated that there was no significant difference between PD and control brains (average 21±5%, t = 0.68, p = 0.5) (Figure 3D). As Ndfip1 has been linked to the regulation of DMT1 [5], [7], we sort to identify the expression of DMT1 in dopaminergic neurons. Peroxidase staining revealed DMT1 expression in the cytoplasm of dopaminergic neurons (Figure 3E). To further define the relationship between Ndfip1 and DMT1 we also conducted double labelling experiments using fluorescent staining, however, we were unable to achieve reliable staining for DMT1 using this technique.

**Figure 4:**
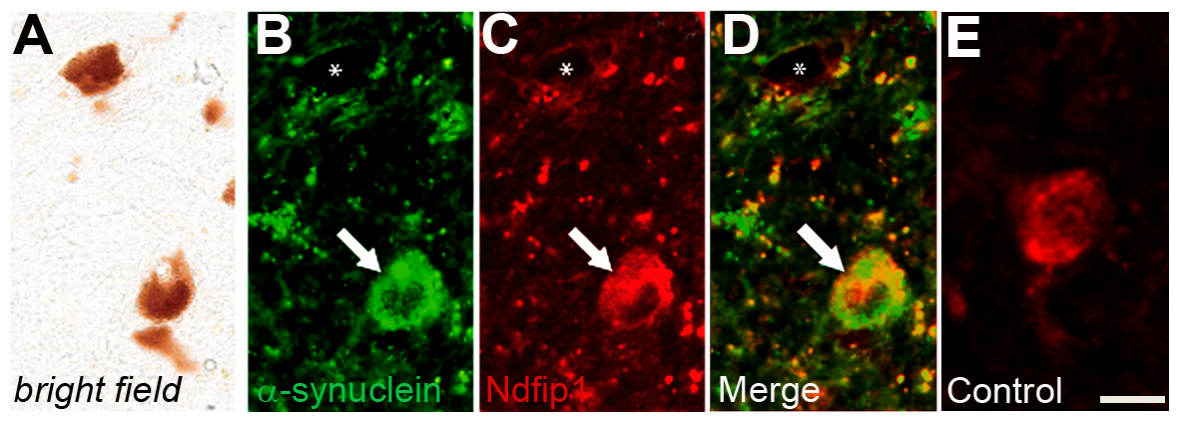
Ndfip1 is expressed in dopaminergic neurons containing α-synuclein deposits. (A) Bright field image of dopaminergic neurons in the substantia nigra of a PD brain. (B–D) Fluorescent labelling of α-synuclein and Ndfip1 from the bright field image shows deposits of α-synuclein which co-label with Ndfip1 in a dopaminergic neuron (arrow). Asterisk marks a dopaminergic neuron with neither α-synuclein nor Ndfip1 positive labelling. (E) Fluorescent labelling of Ndfip1 in a control brain. Scale bar: 25 µm.

Ndfip1 is known to be expressed in response to cellular stress [3], we therefore investigated if Ndfip1 levels were correlated with α-synuclein deposits, a marker of PD-induced stress. Double labelling experiments using Ndfip1 and α-synuclein antibodies were performed on PD brains and we observed that significantly more pigmented neurons with α-synuclein deposits contained Ndfip1 immunoreactivity (average 54±8%, t = 5.35, p = 0.006, versus 21±5% for total dopaminergic neurons) (Figure 4). These findings indicate that Ndfip1 is expressed in dopaminergic neurons of the substantia nigra and that cells undergoing stress, as indicated by α-synuclein deposits, can upregulate the expression of Ndfip1.

### Ndfip1 is misexpressed in astrocytes in Parkinson's disease

**Figure 5:**
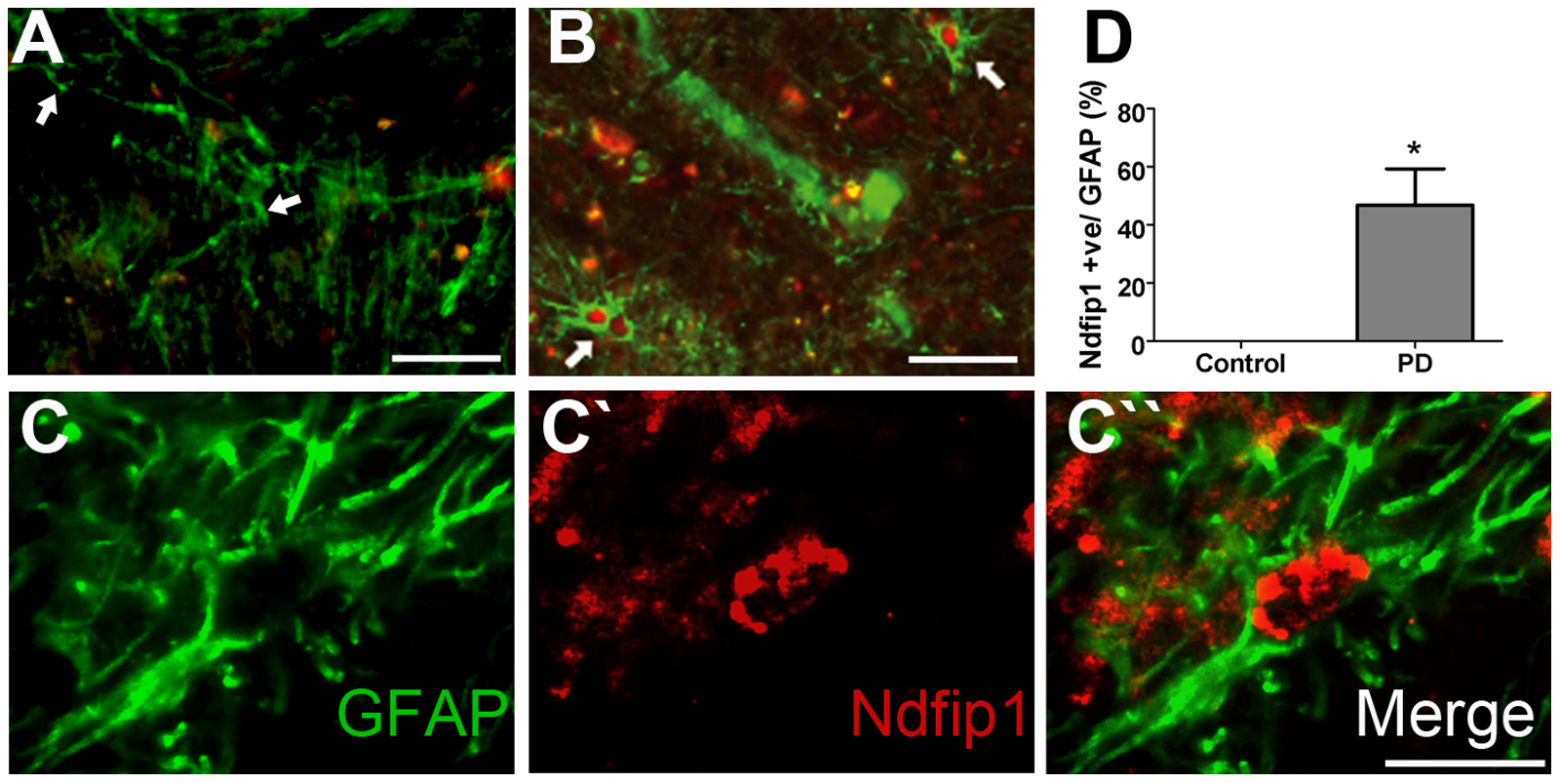
Ndfip1 is expressed in GFAP positive cells of the substantia nigra in PD. (A) Low power image of control brain showing no co-labelling of Ndfip1 (red) or GFAP (green) in the same cell (arrows). (B) Low power image of a PD brain showing co-labelling of Ndfip1 (red) with GFAP (green) in the same cell (arrows). (C-C″) High power image shows Ndfip1 (red) and GFAP (green) positive staining in the substantia nigra of the PD brain. (D) Quantification of GFAP-immunopositive cells co-labelled with Ndfip1 in substantia nigra of both control and PD brains. (D) Values are the mean ±SD, * p<0.05 Mann Whitney U tests. Scale bars: 50 µm (A, B), 10 µm (C).

The biochemical analysis of PD brains indicated that Ndfip1 levels were increased in the substantia nigra in response to the disease state, however our immunohistochemical analysis of PD brains did not show a significant increase in Ndfip1 positive dopaminergic neurons of the substantia nigra when compared to controls. Rather, Ndfip1 upregulation in dopaminergic neurons was correlated with disease stress associated with α-synuclein deposits. To further identify cell types that may express Ndfip1 in PD brains we performed double labelling experiments with GFAP to mark astrocytes within the substantia nigra. In control brains, we observed no double labelling with Ndfip1 and GFAP (Figure 5A), consistent with our previous findings in rodents where Ndfip1 is not normally found in astrocytes [3], or in embryonic human brains where only neurons were found to express Ndfip1 [5]. In contrast, we found in PD a positive correlation between GFAP-positive astrocytes and immunoreactivity for Ndfip1 (t = 3.8, p = 0.013) (Figure 5B–D). These findings suggest that a fraction of the increased Ndfip1 expression in the substantia nigra in PD is a result of Ndfip1 upregulation in astrocytes and confirms a link between Ndfip1 upregulation and PD. However the disease process appears to involve Ndfip1 expression in certain neurons and also in certain astrocytes with unknown disease attributes.

## Discussion

In PD, dopaminergic neurons of the substantia nigra are preferentially susceptible to the misregulation of iron as the metabolism of dopamine gives rise to various molecules that can act as endogenous toxins in the presence of iron [24], [25]. The resulting oxidative stress can inhibit proteasome function [26] and result in the formation of protein aggregates, a hallmark of PD pathology. It is therefore of interest to determine if dopaminergic neurons have protective pathways against metal toxicity. In this study, we report the upregulation of the E3 ligase adaptor protein Ndfip1 in PD brains. Ndfip1 upregulation was correlated with increased iron levels and we observed significant increased expression in a subset of dopaminergic neurons that contain α-synuclein deposits as well as astrocytes in PD brains. As many astrocytes also abnormally contain α-synuclein in PD-affected regions [27], the increased Ndfip1 expression appears directly associated with the abnormal accumulation of α-synuclein in PD. In rodent injury models Ndfip1 has been identified as a neuroprotectant due to its upregulation in surviving neurons following stress caused through stroke or traumatic brain injury [3], [4], [6]. Ndfip1 can be upregulated in response to extracellular stress such as hypoxia or metal toxicity [4], [5], and this activation results in the regulation of a number of critical proteins such as DMT1. Previously we have observed that in an environment where metal concentrations are elevated, Ndfip1 is upregulated by a mechanism that does not require either HSP70 or HIF-1α response [28], and resulted in the binding and degradation of DMT1 [5]. It should be noted that DMT1 abundance can also be regulated transcriptionally and differential expression of DMT1 isoforms having been observed due to iron exposure [10], hypoxia [29] and NFκB activation [30].

In the present report, we observed a protective function for Ndfip1 in mouse dopaminergic neurons where loss of Ndfip1 was found to increase dopaminergic neuron susceptibility to iron induced death. These results suggested that in PD Ndfip1 was upregulated in the substantia nigra as a stress response to protect cells against rising metal toxicity due to failing metal homeostasis. Indeed, our biochemical results from whole tissue lysates confirmed raised Ndfip1 levels in PD brains compared to controls, supporting our overall hypothesis. However, immunohistochemical staining of dopaminergic neurons showed no overall increase in the number of neurons containing Ndfip1 compared to controls. Instead, we found a significant increase in dopaminergic neurons containing both α-synuclein deposits and upregulated Ndfip1, suggesting heterogeneous patterns of Ndfip1 expression in dopaminergic neurons of the substantia nigra. The accumulation of α-synuclein has been shown to be secondary to iron concentrations, resulting in the formation of Lewy bodies [31], suggesting a link between iron concentrations and Ndfip1 observed in neurons containing α-synuclein deposits. Thus neurons containing both α-synuclein deposits and Ndfip1 may represent defensive snapshots of neurons undergoing metal stress. Due to the important association between metal accumulation and protein aggregation in PD, we tried to identify the relationship between Ndfip1 and DMT1 in PD brains. Despite extensive efforts we were unable to obtain reliable fluorescent staining for DMT1 in human tissue and could only identify its expression in dopaminergic neurons using peroxidase-conjugated antibody techniques. This technical limitation restricts our current understanding for the role of Ndfip1 in regulating DMT1 levels in the PD brain.

The pattern of Ndfip1 upregulation in the PD substantia nigra holds parallels with Ndfip1 activation in cortical injury [3]. In rodents after traumatic brain injury or stroke, Ndfip1 upregulation is maximal in surviving neurons at the lesion periphery rather than the core where most cells are necrotic. On this basis, we postulate that neurons demonstrating Ndfip1 upregulation were alive pre-mortem, albeit in a compromised state. However, this comparison needs to take into account that PD is a degenerative disease (unlike trauma and stroke which are acute events), therefore Ndfip1 upregulation here might also serve totally different functions. For instance, failure of the ubiquitin proteasome system is linked to PD pathology [32], therefore Ndfip1 upregulation may represent a reactive response to further accelerate Nedd4-mediated ubiquitination for substrate trafficking or degradation. Alternatively, Ndfip1 response could result in the ubiquitination of DMT1, but failure of the ubiquitin proteosome system may prevent efficient degradation of the metal transporter. It should also be noted that Ndfip1 has a number of other protein targets, including PTEN a major tumor suppressor protein which has roles in cell survival pathways [4], [33], as such the role of Ndfip1 in PD could be multifaceted and involve divergent pathways within the cell. These questions require testing using genetic tools in rodent models of PD.

Finally, the expression of Ndfip1 in astrocytes of PD brains is an unexpected finding given that in healthy brains, Ndfip1 has never been encountered in non-neuronal elements in either the forebrain or midbrain. A number of explanations need to be considered for this response. It is possible that *de novo* Ndfip1 expression occurs in astrocytes in response to the disease process, and in particular to abnormal α-synuclein accumulation. The role that astrocytes play in PD is currently debated, but it is known that astrocytes can accumulate α-synuclein causing the recruitment of phagocytic microglia that can attack neurons in selected brain regions [34]. The mechanism of Ndfip1 activation and function in astrocytes during PD is not clear, however astrocytes are also known to express DMT1 and it is possible that Ndfip1 in astrocytes has similar functions as in neurons, to regulate DMT1 and limit the entry of metals.

In conclusion, we report that Ndfip1 is upregulated in dopaminergic neurons undergoing stress in the substantia nigra, the region of major neuronal loss in PD patients. Unexpectedly, Ndfip1 was also upregulated in astrocytes, a cell type not previously known to express the protein. The link between increased iron levels in PD and Ndfip1 function, suggests that DMT1 ubiquitination by Ndfip1/Nedd4-system may be involved in cell protection pathways to prevent metal toxicity.
